# The reliability of postural balance measures in single and dual tasking in elderly fallers and non-fallers

**DOI:** 10.1186/1471-2474-9-162

**Published:** 2008-12-09

**Authors:** Jaap Swanenburg, Eling D de Bruin, Kathrin Favero, Daniel Uebelhart, Theo Mulder

**Affiliations:** 1 Department of Rheumatology and Institute of Physical Medicine, University Hospital Zurich, Zurich, Switzerland; 2 Institute of Human Movement Sciences and Sport, ETH, Zurich, Switzerland; 3 Centre of Osteoporosis, University Hospital Zurich, Zurich, Switzerland; 4 Royal Netherlands Academy of Arts and Sciences, Amsterdam, The Netherlands; 5 Center for Human Movement Sciences, University Medical Center Groningen, Groningen, The Netherlands

## Abstract

**Background:**

The purpose of this study was to determine the reliability of a forceplate postural balance protocol in a group of elderly fallers and non-fallers. The measurements were tested in single and dual-task conditions, with and without vision.

**Methods:**

37 elderly (mean age 73 ± 6 years) community-dwellers were included in this study. All were tested in a single (two-legged stance) and in a dual-task (two-legged stance while counting backwards aloud in steps of 7's) condition, with and without vision. A forceplate was used for registering postural variables: the maximal and the root-mean-square amplitude in medio-lateral (Max-ML, RMS-ML) and antero-posterior (Max-AP, RMS-AP) direction, mean velocity (MV), and the area of the 95% confidence ellipse (AoE). Reliability of the test protocol was expressed with intraclass correlation coefficients (ICC), with 95% limits of agreement (LoA), and with the smallest detectable difference (SDD).

**Results:**

The ICCs for inter-rater reliability and test-retest reliability of the balance variables were r = 0.70–0.89. For the variables Max-AP and RMS-AP the ICCs were r = 0.52–0.74. The SDD values were for variable Max-ML and Max-AP between 0.37 cm and 0.83 cm, for MV between 0.48 cm/s and 1.2 cm/s and for AoE between 1.48 cm^2 ^and 3.75 cm^2^. The LoA analysis by Bland-Altman plots showed no systematic differences between test-retest measurements.

**Conclusion:**

The study showed good reliability results for group assessment and no systematic errors of the measurement protocol in measuring postural balance in the elderly in a single-task and dual-task condition.

## Background

Various balance tests [1,2] and measurements [3-7] have been developed and presented to obtain appropriate information of balance capabilities during standing. Although tests for postural control with functional balance scales are easy to perform and are suitable for daily clinical use they often lack accuracy. Technology based laboratory systems may give more detailed information about postural balance [8], but are often difficult to use in a clinical setting.

Quantitative posturography is a frequently used technique for measuring postural control [9]. This technique covers all force platforms used to quantify postural control in upright stance in either static or dynamic conditions. The employed force platform indirectly detects changes of postural sway by assessing the ground-reaction forces. These ground-reaction forces are used to calculate the centre of pressure (COP), which reflects the trajectory of the centre of mass and the torque acting on the surface [10]. Various balance variables can be derived from the COP movement, e.g. the root mean square (RMS) of COP amplitudes in anterior-posterior and medio-lateral direction or the maximum COP displacement in anterior-posterior and medio-lateral direction [11-15]. It is assumed that these measures relate to impaired postural control in humans. However, in spite of the frequent use of these measures only a small number of studies have reported on the reliability of postural balance measures [12,13,15-17].

Commonly identified flaws in reliability studies are the exclusive use of healthy individuals, questionable applicability in clinical practice, low sample size, the absence of a protocol and the use of inadequate statistics [18]. It is questionable whether the test results of healthy elderly for example can be generalized to specific sub-populations, e.g. fallers, in clinical practice. Only very few studies tested the reliability of postural assessment with a force platform in patient groups. Benvenuti and colleagues (1999) assessed patients with a variety of chronic pathologic conditions resulting in balance problems; however, they did not specifically focus on fallers or non-fallers [16]. Stroke survivors and patients suffering from diabetic neuropathy were assessed by Corriveau and colleagues (2001) but these authors excluded subjects if they reported visual or somatosensory impairments or reported at least 1 fall in the past year [17]. The same exclusion of fallers was performed by Lafond et al. (2004) [15].

There seems to be a need to perform reliability assessments of postural control in groups with identified fallers and non-fallers. No reliability studies have been reported that specifically included fallers. However, since one-third of community-dwelling people over 65 years of age experience one or more falls each year, it seems important to include elderly fallers in reliability studies [18-22].

The applicability of test measures in clinical practice is another important point to consider. Most reliability studies used single-task procedures consisting of standing quietly while manipulating the visual input and/or changing the base of support (BOS). Mulder et al. (2002) argued that although a motor system may deteriorate across time, many assessment procedures show no changes in performance. The authors state that this phenomenon is related to the fact that the level of functional reorganization of a (changing) motor system is not necessarily reflected in the 'pure' end-result of a task, but might be reflected also in the increasing compensatory costs across time [23]. This would mean that assessment procedures that are used in clinical practice should also be sensitive to this phenomenon when we want to be able to detect possible underlying pathologies. In other words the compensatory costs, necessary to keep the motor output optimal, should be estimated in clinical protocols.

The basic idea behind the dual-task methodology is that the performance of a difficult (non-automated) task interferes with other simultaneously performed tasks [24]. Hence, by employing an attention demanding task, it is possible to use the degree of interference of this task with the primary task (e.g. standing) as a measure of the attention demands (cognitive compensation) of the primary task. There is indeed a growing use observable of dual-task procedures in studies focusing on recovery after damage to the motor system [25] or in neurological assessment [26]. However, there are not many studies that include reports on the reliability of the used protocols. We found only one study that focused on the reliability of the postural measures and that used a simultaneous secondary task during performance of the primary (postural) task [4]. At the same time it has been reported that falls seem to occur frequently during activities in which attention has to be divided between two tasks [27]. This observation further underscores the potential value and necessity of dual task testing. Furthermore, because of inconsistencies in the design and analysis of method evaluation studies, a high proportion of prognostic studies were presented with poor methodology which resulted in the presentation of conflicting interpretation of variability of the measures. This led the Work Package 3 of the Prevention of Falls Network Europe to formulate criteria for evaluation of measurement properties of clinical balance measures for fall prevention studies [28]. The purpose of the present study was, therefore, to determine the interrater and test-retest reliability of quantitative postural control measures in elderly fallers and non-fallers, tested under single and dual-task conditions, with and without vision, and considering both relative and absolute reliability.

## Methods

### Participants

Thirty-seven community dwellers participated in the study (29 women), the average age was 73 ± 6 years (range 61–85 years). The inclusion criteria were fallers and non-fallers older than 60 years of age of both genders. Exclusion criteria were participants who were unable to understand (language) the purpose of the study, severe psychological or psychiatric problems, chronic substance-abuse (medication, drugs and/or alcohol), and patients under chronic therapy with neuroleptics, sedatives, anti-epileptics and anti-depressives. A structured interview that considered recommendations on falls outcome measures [29] was used to assess the numbers of falls in the previous year. A fall was defined as any event that caused unintentional contact by the torso or upper limbs to the ground or to some lower level, other than as a consequence of a violent blow, loss of consciousness, or a sudden onset of paralysis as in stroke or epileptic seizure [30]. A faller was defined in this study as a subject that sustained more than one fall within the last 12 months. The measurements took place at the Institute of Physical Medicine (Department of Rheumatology), University Hospital Zurich. All participants gave their informed written consent and were blinded to the purpose of the measurements. The study was approved by the local ethics committee.

### Experimental procedure

The AMTI Accusway system for balance and postural sway measurement (Advanced Mechanical Technology, Inc., Watertown, Massachusetts) was used for collecting the data. The Accusway system consists of a portable force platform and SWAYWIN software for data acquisition and analysis. The system measures ground reacting force and moments in 3 orthogonal directions with a sampling frequency of 50 Hz. These provide the COP coordinates, which enables the calculation of the maximum displacement in the anterior-posterior and medial-lateral direction (Max-AP; Max-ML), the root-mean-square amplitude in anterior-posterior and medial-lateral direction from the centroid in x- and y-axis (RMS-AP; RMS-ML), the mean velocity (MV) and the area of the 95^th ^percentile ellipse (AoE).

Before the measurements took place, the balance platform was strapped with an anti-slip plastic cover (1 mm). The participant then took a comfortable barefooted, double-legged stance on the platform. Because changes in the Base of Support (BOS) have a substantial effect on postural control [14]; the outlines of both feet were marked on the plastic cover with a permanent marker in order to obtain standardised individual foot positions for the repeated measurements. After leaving the platform, the individual's BOS was entered in the Accusway Plus system [31]. Maximal BOS width and hip width, measured at the major trochanter femoris, were recorded with an anthropometric calliper (Lafayette Instrument Company, Lafayette, IN).

### Measurement Design

The participants were tested individually within a single session that lasted about 25 minutes. First, instructions of the cognitive task were given, followed by a full performance of the cognitive tasks while seated. Thereafter, the participants were instructed to stand on the pre-marked plastic cover with the arms by the sides and eyes open while looking straight ahead.

The postural balance measurements were collected under two task conditions: standing quiet (without a secondary cognitive task) and standing quiet combined with counting backwards in steps of seven. Each task consisted of 4 trials and the average of the 4 trials was taken to obtain a reliable measure [17]. Each separate trial lasted 20 seconds, followed by a break of 20 seconds [32].

The total 20 seconds of the trial was used for the calculations. Between each task, the participants were allowed to sit down for a 2-minute break. Both tasks were measured with and without vision. The order of tasks (single, dual, with and without vision) was changed randomly to control for the effects of fatigue and learning. The rationale for this procedure was primarily based on the fact that the duration of a trial in quiet standing is limited due to fatigue, particularly in pathologic elderly [15]. Furthermore, the optimum test-retest reliability for our protocol was assumed to be obtained at 20s trial durations [32], and we wanted a test that is feasible to be implemented in a clinical setting where time constraints play an important role.

### Cognitive task

Counting backwards, as a cognitive task, showed significant degradation in postural stability in healthy adults and healthy elderly [33-35]. Therefore counting backwards in steps of 7's was also used as additional task in the present study. The participant was asked to count back as fast and accurate as possible in 20 seconds [36,37]. If the counting backwards in steps of sevens was too difficult, steps of threes or ones were used instead. The starting number was selected at random from a range of 80–99. For those participants who were able to count back to zero within 20 seconds a starting number was selected within the range of 121 and 199. The counting was controlled continuously for accuracy and every mistake was noted. No feedback on performance was given during the testing. Evaluation of performance during the cognitive task included the difficulty (sevens, threes or ones) of subtraction units and the number of mistakes made by the participant during calculation.

To evaluate the performance of the cognitive task the difficulty (sevens, threes or ones) of subtraction and the number of mistakes made by the participant during the calculation were used to define 6 performance scores (Cognitive Difficulty Score, CDS). The lowest score is designated number 1 and is given when mistakes are made during counting backwards in ones. The highest score (6) is given when counting backward in sevens is possible without making mistakes. With increasing numerical complexity the CDS is increasing. An overall group score (GS) was calculated by taking a mean of all individual scores (Table 1).

**Table 1:** Cognitive Difficulty Score, taking in account task difficulty and mistakes made during dual tasking

**Difficulty Counting Backwards**	**Mistakes Made**	**Cognitive Difficulty Score**
1	Yes	1
1	No	2

3	Yes	3
3	No	4

7	Yes	5
7	No	6

### Visual conditions

The two tasks were tested under two different visual conditions:

a) Normal vision; the participants were instructed to view a fixed grey cross; the arms of the cross were 1 meter long and aligned horizontal. The vertical arms were 0.5 meter long. The cross was located in the middle of a screen (1.5 m × 1.5 m), which was positioned 2 meters in front of the forceplate. The height of the grey cross was fixed at 1.5 m. All participants used their own glasses when needed, to have optimal individual visual acuity.

b) Vision was occluded with a pair of custom-made opaque goggles that prevented the subject from perceiving visual information without blocking the light in general. The participants were instructed to keep their eyes open inside the goggles.

### Reproducibility Protocol

**Figure 1:**
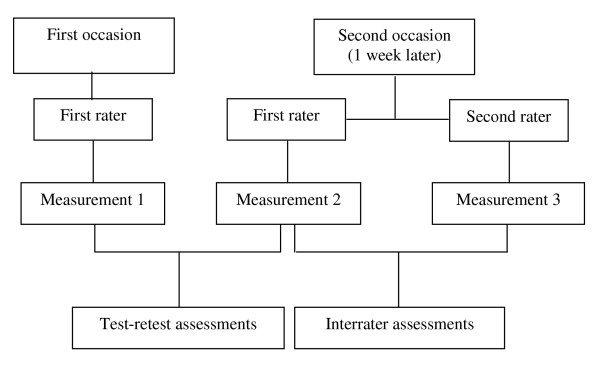
Flow diagram of assessments timeline

For the test-retest study, all participants were evaluated by the first rater on 2 occasions with an inter-measurement interval of 7 days. Both measurements were performed at the same time of the day in the same measurement room. Additionally at the second measurement occasion the second rater performed a third measurement to evaluate the interrater reliability. The order of the rater was changed after each participant (Figure 1).

### Statistical Analysis

Descriptive statistics were used to describe the participant's characteristics. The one-sample Kolmogorov-Smirnov test was used to check the normality of the distributions.

De Vet and colleagues (2006) recently suggested using both reliability and agreement parameters in reliability studies because this allows gaining a better insight on the performance of measuring a variable [38]. Reliability parameters assess whether a measurement device can distinguish between groups of patients and between individual patients [39]. Agreement parameters measure the ability to achieve the same value in two measurements, and thus give an indication of the size of the measurement errors [40].

#### Reliability parameters

The intraclass correlation coefficient (ICC) was used as a parameter of reliability. The ICC_(2,1) _model was selected to test the interrater reliability, and the ICC_(3,1) _model to estimate the test-retest reliability [41,42].

#### Agreement parameters

For both the test-retest and the interrater reliability the smallest detectable differences (SDD) were determined by the 95% SEM (1.96 √ 2 √ SEM; SEM = √mean square error) [43,44].

The 95% limits of agreement (LoA) were for both the test-retest and the interrater reliability assessed according Bland and Altman. LoA was calculated by: mean of the differences ± 1.96*SD. LoA indicates the total error, which is systematic error and random error combined. Discrepancies between measurements were also assessed by visual interpretations of the amount of agreement of the means of two trials against the difference between the trials (Bland and Altman Plots). The use of 95% confidence intervals of the range of differences between the two trials demonstrates how close the measurements agree on different occasions. All calculations were considered as significant at the 5% confidence level [45].

The data were entered, stored, and analysed in SPSS 12.0.1 statistical software (SPSS, Inc., Chicago, IL).

## Results

A total of 37 participants were recruited (29 women), the average age was 73 ± 6 years (range 61–85 years) and a total of 11 fallers were identified. The participant's characteristics are shown in Table 2. All participants were able to count backward in steps of sevens. A total of 20 participants made counting mistakes, whereas 17 made no mistakes. The group score (GS) of first rater measurements in both occasions was 5.5. The second rater reported a GS of 5.4 within his measurement (maximal GS possible is 6; see Table 3). There was no significant difference in GS between the raters.

**Table 2:** Participants' characteristics

	**All (n = 37)**	**Non-fallers (n = 26)**	**Fallers (n = 11)**	**P**
Female	29	18	11	
Male	8	8	0	
Age; years (SD)	73 (6)	71 (6)	76 (4)	ns.
Range	61/85	61/85	67/83	
Weight; kg (SD)	67 (11)	69 (11)	64 (12)	ns.
Height; cm (SD)	165 (7)	166 (8)	161 (5)	**0.05**
Hip width (cm)	34.2(3)	34.4(2)	33.7(4)	ns.
BOS width (cm)	27.7(4)	29.4(3)	30.5(4)	ns.

SD = Standard Deviation; p = unpaired t-tests; *p < 0.05 fallers and non-fallers; ns = not significant, BOS = base of support

**Table 3:** Results of the group scores of Cognitive Difficulty Score, taking in account task difficulty and mistakes made during dual tasking.

	**All (n = 37)**	**Non-fallers (n = 26)**	**Fallers (n = 11)**	**p**
Group Score (GS)				
Rater 1 first occasion	5.5	5.5	5.4	ns.
Rater 1 second occasion	5.5	5.5	5.3	ns.
Rater 2 second occasion	5.4	5.4	5.4	ns.

p = unpaired t-tests; *p < 0.05 fallers and non-fallers; ns = not significant,

### Reliability parameters

Two postural balance variables, which had no normal distribution, were log transformed and marked (see in Table 4 and Table 5). Our study showed good ICC values of the postural balance measurement protocol, e.g. test retest, as well as for interrater reliability. The ICC(_2.1_) for interrater reproducibility and the ICC(_3.1_) for test-retest reproducibility of the balance variables Max-ML, RMS-ML, MV and AoE were ranging between r = 0.70–0.89. For the variables 'Max-AP' and 'RMS-AP' the ICCs ranged between r = 0.52–0.74. The results of the interrater ICCs are summarised in Table 4 and the results of the test-retest are presented in Table 5.

**Table 4:** Interrater Reliability Parameters

Balance-Variable	Vision	Task	All n = 37	Non-Faller n = 26	Faller n = 11
			**Interrater ICC **95% CI	**Interrater ICC **95% CI	**Interrater ICC **95% CI
Max-ML (cm)	Vision	Single	**0.76 **0.60/0.87	**0.80 **0.60/0.90	**0.72 **0.28/0.91
		Dual	**0.75 **0.56/0.86	**0.84 **0.68/0.93	**0.30 **-0.38/0.75
	No-Vision	Single	**0.73 **0.53/0.85	**0.78 **0.57/0.90	**0.55 **0.01/0.85
		Dual	**0.72 **0.52/0.84	**0.72 **0.47/0.82	**0.70 **0.32/0.93

RMS-ML (cm)	Vision	Single	**0.84 **0.71/0.92	**0.83 **0.75/0.92	**0.89 **0.68/0.97
		Dual	**0.71 **0.51/0.84	**0.75 **0.51/0.88	**0.45 **-0.22/0.82
	No-Vision	Single	**0.70 **0.48/0.83	**0.72 **0.47/0.87	**0.62 **0.12/0.88
		Dual	**0.86 **075/0.93	**0.87 **073/0.94	**0.83 **0.47/0.895

Max-AP (cm)	Vision	Single	**0.52 **0.24/0.72	**0.56 **0.24/0.72	**0.47 **-0.07/0.81
		Dual	**0.64 **0.40/0.80	**0.61 **0.38/0.80	**0.68 **0.31/0.94
	No-Vision	Single	**0.74 **0.55/0.86	**0.84 **0.67/0.92	**0.40 **-0.21/0.92
		Dual	**0.70 **0.48/0.83	**0.72 **0.46/0.86	**0.64 **0.12/0.89

RMS-AP (cm)	Vision	Single	**0.57 **0.30/0.75	**0.42 **0.05/0.69	**0.77 **0.37/0.93
		Dual	**0.46 **0.16/0.68	**0.40 **0.13/0.68	**0.77 **0.29/0.93
	No-Vision	Single	**0.73 **0.54/0.85	**0.85 **0.69/0.93	**0.45 **-0.08/0.80
		Dual	**0.72 **0.51/0.84	**0.75 **0.51/0.88	**0.60 **0.04/0.88

MV (cm/s)	Vision	Single	**0.76 **0.57/0.87	**0.81 **0.57/0.87	**0.70 **0.20/0.91
		Dual	**0.85 **0.72/0.92	**0.80 **0.60/0.90	**0.95 **0.82/0.98
	No-Vision	Single*	**0.87 **0.77/0.93	**0.89 **0.77/0.95	**0.78 **0.33/0.93
		Dual	0.84 0.72/0.92	**0.85 **0.70/0.93	**0.83 **0.51/0.95

AoE (cm^2^)	Vision	Single	**0.65 **0.42/0.81	**0.65 **0.35/0.83	**0.69 **0.22/0.90
		Dual*	**0.74 **0.54/0.85	**0.75 **0.51/0.88	**0.57 **-0.02/0.86
	No-Vision	Single	**0.66 **0.43/0.81	**0.76 **0.54/0.89	**0.67 **-0.04/0.79
		Dual	**0.81 **0.66/0.90	**0.83 **0.65/0.92	**0.69 **0.17/0.91

* = log transformed

**Table 5:** Test-Retest Reliability Parameters

			All n = 37	Non-Faller n = 26	Faller n = 11
Balance-Variable	Vision	Task			
			**Test-retest ICC **95% CI	**Test-retest ICC **95% CI	**Test-retest ICC **95% CI
Max-ML (cm)	Vision	Single	**0.77 **0.60/0.88	**0.75 **0.52/0.88	**0.71 **0.46/0.95
		Dual	**0.71 **0.51/0.84	**0.80 **0.51/0.91	**0.32 **-0.31/0.76
	No-Vision	Single	**0.75 **0.56/0.86	**0.83 **0.65/0.92	**0.53 **-0.07/0.85
		Dual	**0.77 **0.59/0.87	**0.81 **0.62/0.91	**0.59 **0.03/0.87

RMS-ML (cm)	Vision	Single	**0.79 **0.63/0.89	**0.73 **0.48/0.87	**0.71 **0.55/0.86
		Dual	**0.75 **0.57/0.86	**0.80 **0.60/0.90	**0.58 **0.01/0.85
	No-Vision	Single	**0.72 **0.51/0.84	**0.71 **0.45/0.86	**0.69 **0.33/0.93
		Dual	**0.85 **0.73/0.92	**0.88 **0.75/0.94	**0.86 **0.15/0.90

Max-AP (cm)	Vision	Single	**0.55 **0.28/0.74	**0.43 **0.06/0.70	**0.55 **-0.04/0.85
		Dual	**0.63 **0.39/80	**0.56 **0.22/0.77	**0.57 **0.22/0.77
	No-Vision	Single	**0.64 **0.41/0.80	**0.83 **0.65/0.92	**0.43 **-0.19/0.81
		Dual	**0.61 **0.35/0.78	**0.68 **0.40/0.84	**0.40 **-0.22/0.79

RMS-AP (cm)	Vision	Single	**0.51 **0.23/0.71	**0.31 **-0.08/0.62	**0.77 **0.36/0.89
		Dual	**0.54 **0.27/0.74	**0.55 **0.21/0.77	**0.50 **-0.11/0.83
	No-Vision	Single	**0.69 **0.47/0.83	**0.86 **0.72/0.94	**0.39 **-0.24/0.80
		Dual	**0.58 **0.31/0.76	**0.64 **0.35/0.82	**0.38 **-0.25/0.79

MV (cm/s)	Vision	Single	**0.89 **0.79/0.94	**0.84 **0.68/0.93	**0.81 **0.56/0.97
		Dual	**0.84 **0.71/0.91	**0.82 **0.64/0.92	**0.82 **0.34/0.88
	No-Vision	Single*	**0.89 **0.80/0.94	**0.87 **0.74/0.94	**0.81 **0.71/0.98
		Dual	**0.79 **0.63/0.89	**0.78 **0.55/0.89	**0.75 **0.54/0.96

AoE (cm^2^)	Vision	Single	**0.75 **0.57/0.86	**0.62 **0.32/0.81	**0.81 **0.63/0.98
		Dual*	**0.79 **0.63/0.89	**0.81 **0.62/0.91	**0.49 **-0.11/0.83
	No-Vision	Single	**0.70 **0.50/0.84	**0.73 **0.49/0.87	**0.60 **0.04/0.87
		Dual	**0.76 **0.57/0.87	**0.80 **0.61/0.91	**0.64 **-0.15/0.82

* = log transformed

### Agreement parameters

The SDD values for variables Max-ML and Max-AP were 0.37 cm and 0.83 cm respectively. Variable MV lay between 0.48 cm/s and 1.2 cm/s. For variable AoE the SDD values were between 1.48 cm^2 ^and 3.75 cm^2 ^(see Table 4). To detect change in clinical practice beyond measurement error potential changes should be larger than these SDD values.

The LoA showed very small systematic error between test-retest and interrater agreement. The mean of the differences for variable Max-ML and Max-AP were between 0.0 cm and 0.08 cm. For variable MV between 0.03 cm/s and 0.18 cm/s and for variable AoE between 0.06 cm^2 ^and 0.51^2 ^(see Table 6).

**Table 6:** Agreement parameters

	Vision	Task	Rater 1/1st Mean (SD)	Rater 1/2nd Mean (SD)	Rater 2 Mean (SD)	Test -retest LoA	Test-retest SDD	Interrater LoA	Inter-rater SDD
Max-ML (cm)	Vision	Single	0.61 (0.28)	0.61 (0.28)	0.57 (0.24)	0.00 ± 0.37	0.37	0.04 ± 0.34	0.34
		Dual	0.95 (0.65)	0.89 (0.48)	0.89 (0.54)	0.06 ± 0.85	0.85	0.00 ± 0.72	0.72
	No-Vision	Single	0.69 (0.30)	0.64 (0.23)	0.66 (0.26)	0.05 ± 0.38	0.37	-0.03 ± 0.35	0.35
		Dual	0.92 (0.43)	0.86 (0.41)	0.80 (0.37)	0.06 ± 0.56	0.56	0.06 ± 0.57	0.57

RMS-ML (cm)	Vision	Single	0.24 (0.09)	0.25 (0.10)	0.23 (0.09)	-0.01 ± 0.13	0.12	0.02 ± 0.10	0.09
		Dual	0.39 (0.25)	0.35 (0.16)	0.37 (0.26)	0.03 ± 0.29	0.29	-0.02 ± 0.31	0.32
	No-Vision	Single	0.29 (0.15)	0.26 (0.10)	0.26 (0.10)	0.03 ± 0.18	0.18	0.00 ± 0.15	0.15
		Dual	0.36 (0.17)	0.34 (0.17)	0.34 (0.21)	0.02 ± 0.18	0.18	0.00 ± 0.20	0.20

Max-AP (cm)	Vision	Single	0.85 (0.25)	0.84 (0.33)	0.83 (0.21)	0.01 ± 0.55	0.55	0.01 ± 0.53	0.53
		Dual	1.20 (0.52)	1.15 (0.44)	1.12 (0.39)	0.05 ± 0.81	0.81	0.03 ± 0.70	0.70
	No-Vision	Single	1.11 (0.48)	1.04 (0.35)	1.07 (0.32)	0.07 ± 0.70	0.70	-0.03 ± 0.48	0.48
		Dual	1.35 (0.59)	1.27 (0.39)	1.18 (0.35)	0.08 ± 0.87	0.83	0.10 ± 0.55	0.55

RMS-AP (cm)	Vision	Single	0.36 (0.09)	0.34 (0.12)	0.34 (0.09)	0.01 ± 0.21	0.21	0.00 ± 0.19	0.20
		Dual	0.46 (0.18)	0.45 (0.15)	0.45 (0.16)	0.00 ± 0.31	0.30	0.00 ± 0.32	0.32
	No-Vision	Single	0.44 (0.15)	0.41 (0.12)	0.43 (0.11)	0.03 ± 0.20	0.20	-0.02 ± 0.16	0.15
		Dual	0.50 (0.20)	0.48 (0.13)	0.45 (0.13)	0.02 ± 0.30	0.29	0.03 ± 0.18	0.18

MV (cm/s)	Vision	Single	1.34 (0.57)	1.31 (0.47)	1.24 (0.35)	0.03 ± 0.48	0.48	0.08 ± 0.55	0.55
		Dual	1.83 (0.74)	1.75 (0.60)	1.76 (0.64)	0.08 ± 0.75	0.75	-0.01 ± 0.69	0.68
	No-Vision	Single	1.82 (0.91)	1.64 (0.64)	1.73 (0.86)	0.18 ± *0.90	0.98*	-0.09 ± *0.89	0.93*
		Dual	2.22 (1.33)	2.09 (0.74)	2.10 (0.90)	0.14 ± 1.21	1.21	-0.01 ± 0.92	0.92

AoE (cm^2^)	Vision	Single	1.64 (0.99)	1.58 (1.13)	1.46 (0.81)	0.06 ± 1.48	1.48	0.12 ± 1.61	1.61
		Dual	3.78 (4.43)	3.27 (2.54)	3.34 (3.50)	0.51 ± *5.59	2.62*	-0.07 ± *4.25	2.92*
	No-Vision	Single	2.45 (1.87)	2.09 (1.26)	2.21 (1.33)	0.36 ± 2.40	2.40	-0.12 ± 2.10	2.11
		Dual	3.66 (3.17)	3.18 (2.22)	3.00 (2.53)	0.48 ± 3.75	3.75	0.19 ± 2.89	2.89

* = log transformed

Bland-Altman plots indicated that most points lie within the 95% limits of agreement for test-retest measurements. Only 2 to 3 outliers were found within the plots. In all tables the outliers show both positive and negative differences of the mean, which indicates no systematic effect. Balance variables had the smallest 95% limits of agreement when testing in a single task situation with vision. The opposite was found in the dual-task situation with and without vision. The Bland-Altman plots are presented in the additional file [see Additional file 1].

## Discussion

The purpose of this study was to evaluate the reliability of a forceplate postural balance assessment protocol under single and dual-task conditions in elderly fallers and non-fallers.

This study showed good reliability parameters for the total group of participants although in the non-fallers subgroup the values were higher compared to the fallers (see Table 4). Hence, our findings show the relevance of including symptomatic populations in a reliability study as previously was suggested by Hoving and colleagues (2005)[18]. Furthermore, the results of our study are in line with previous studies that included symptomatic populations, e.g. patients suffering from diabetes, neuropathy or stroke survivors [17]. From a clinical perspective our procedure makes sense because we included symptomatic individuals in our sample. This indicates that the results can be generalised to similar populations in clinical settings. It can be expected that a normal population will, similar to our sample, consist of both fallers and non-fallers. This would mean that our results are generalisable to comparable clinical populations.

The ICC values were different for each balance variable that was assessed. Between the test conditions, vision or no-vision and single or dual task, there were differences in ICC values as well (Tables 4 &5). The results were consistently better in the medial lateral direction compared to the moderate ICC values in the anterior posterior direction. From a clinical perspective these results are encouraging. Day and colleagues (1993) have demonstrated that deterioration of balance control in the elderly primarily occurs in the ML direction during quiet stance [46]. When responding to a plate perturbation older adults also frequently step to especially preserve lateral stability [47]. These findings might be an indication that the main focus in assessment should be put on the mediolateral force plate variables. In these cases there are no large differences in reliabilities of the test protocol between vision and no-vision and between single and dual-task testing conditions. Our protocol reveals no large differences in reliability between these test conditions. The most optimal variables that should be assessed when groups of subjects are compared seem to be Max-ML, RMS-ML, and MV since these all show highest ICC values. On an individual subject assessment level the agreement parameters of the Max-ML, RMS-ML, and MV variables seem to be promising too. However, future intervention type studies for individuals should substantiate this assumption.

These results are not in accordance with the results of Corriveau and colleagues (2001), who found better ICC values in the anterior posterior direction than in the medial lateral direction [17]. A possible explanation for these differences could be found in the different assessment protocols used. Our participants were expected to take a comfortable stance position and were expected to repeatedly use this individualised position. This ment that foot position was standardized for each subject, but not across subjects. This was in contrast to Corriveau and colleagues who asked their participants to take a pre-determined stance position of pelvis width.

It is well documented that with increasing stance width a disproportionate reduction in the angular motion about the ankles and feet; e.g. the ankle joint mobility in the frontal plane is reduced with feet apart [48]; can be observed that causes a large reduction in lateral body motion [46]. It is for this reason that we standardised foot positions as previously recommended [50].

The limits of agreement showed no systematic error (bias) between the two measurements of rater 1 (test-retest) or between the measurements of rater 1 and rater 2 (interrater). Our protocol, therefore, seems to be well suited for clinical applications where several clinicians are often responsible for the same kinds of measurement. The resulting SDD values were rather large. At this moment it is difficult to say whether the obtained SDD values are too large to detect clinically meaningful differences on an individual level and would, therefore, be clinically not relevant. SDD values provide information about the size of the error related to a measured value and in the amount of measurement error that should be taken into account when comparing two consecutive measurements. Therefore these SDD values imply to have a rather less satisfactory reliability for assessing individual changes in comparison to group changes. This assumption should be substantiated in further research. It might very well be that the changes caused by interventions are larger, especially in clinical populations, than the SDD found in our study.

With our protocol that has shown to have good reliability in both fallers and non-fallers the next step in research would be to test the validity of this protocol. For that purpose we should perform a prospective study in a group of older individuals that is threatened to fall. It can be argued that in such a measurement design our protocol may have predictive value for subsequent falls.

## Conclusion

In conclusion, our measurement protocol showed good reliability for group assessment with no systematic errors in measuring postural balance in single-task and in dual-task conditions in a group of elderly fallers and non-fallers. These results may form a basis for further research examining, for example, the effects of physical exercise in elderly suffering from balance impairments. The value of the test protocol for individualised assessment remains unclear and should be subject to further research.

## Competing interests

The authors declare that they have no competing interests.

## Authors' contributions

JS designed and performed the study and wrote the manuscript. ED helped to draft the manuscript, monitored the study, and revised the manuscript critically for its content. KF designed and monitored. DU helped with the coordination. TM helped to draft the manuscript and initiated the study. All authors read and approved the final manuscript.

## Pre-publication history

The pre-publication history for this paper can be accessed here:



## Supplementary Material

Additional file 1 **Bland and Altman plots of the measured balance variables**. The data provided represent the analysis with Bland-Altman plots of the measured balance variables. Click here for file

## References

[B1] Mathias S, Nayak US, Isaacs B (1986). Balance in elderly patients: The "Get-up and Go" test. Arch Phys Med Rehabil.

[B2] Berg KO, Wood-Dauphinee S, Williams JI, Gayton D (1989). Measuring balance in the elderly: preliminary development of an instrument. Physiotherapy Canada.

[B3] Juntunen J, Ylikoski J, Ojala M, Matikainen E, Ylikoski M, Vaheri E (1987). Postural body sway and exposure to high-energy impulse noise. Lancet.

[B4] Briggs R, Gossman M, Birch R, Drews J, Shaddeau S (1989). Balance performance among noninstitutionalized elderly women. Phys Ther.

[B5] Wing AM, Clapp S, Burgess-Limerick R (1995). Standing stability in the frontal plane determined by lateral forces applied to the hip. Gait Posture.

[B6] Era P, Schroll M, Ytting H, Gause-Nilsson I, Heikkinen E, Steen B (1996). Postural balance and its sensory – motor correlates in 75-year-old men and women: A cross-national comparative study. J Gerontol A Biol Sci Med Sci.

[B7] Chang H, Krebs DE (1999). Dynamic balance control in elders: Gait initiation assessment as a screening tool. Arch Phys Med Rehabil.

[B8] Alexander NB (1996). Using technology-based techniques to assess postural control and gait in older adults. Clin Geriatr Med.

[B9] Horak FB (1997). Clinical assessment of balance disorders. Gait Posture.

[B10] Goldie PA, Bach TM, Evans OM (1989). Force platform measures for evaluating postural control: reliability and validity. Arch Phys Med Rehabil.

[B11] Stelmach GE, Zelaznik HN, Lowe D (1990). The influence of aging and attentional demands on recovery from postural instability. Aging (Milano).

[B12] Geurts AC, Nienhaus B, Mulder TW (1993). Intrasubject variability of selected force-platform parameters in the quantification of postural control. Arch Phys Med Rehabil.

[B13] Corriveau H, Hebert R, Prince F, Raiche M (2000). Intrasession reliability of the "center of pressure minus center of mass" variable of postural control in the healthy elderly. Arch Phys Med Rehabil.

[B14] Melzer I, Benjuya N, Kaplanski J (2001). Age-Related Changes of Postural Control: Effect of Cognitive Tasks. Gerontology.

[B15] Lafond D, Corriveau H, Hebert R, Prince F (2004). Intrasession reliability of center of pressure measures of postural steadiness in healthy elderly people. Arch Phys Med Rehabil.

[B16] Benvenuti F, Mecacci R, Gineprari I, Bandinelli S, Benvenuti E, Ferrucci L, Baroni A, Rabuffetti M, Hallett M, Dambrosia JM, Stanhope SJ (1999). Kinematic characteristics of standing disequilibrium: reliability and validity of a posturographic protocol. Arch Phys Med Rehabil.

[B17] Corriveau H, Hebert R, Prince F, Raiche M (2001). Postural control in the elderly: an analysis of test-retest and interrater reliability of the COP-COM variable. Arch Phys Med Rehabil.

[B18] Hoving JL, Pool JJ, van Mameren H, Devillé WJ, Assendelft WJ, de Vet HC, de Winter AF, Koes BW, Bouter LM (2005). Reproducibility of cervical range of motion in patients with neck pain. BMC Musculoskelet Disord.

[B19] Hausdorff JM, Edelberg HK, Mitchell SL, Goldberger AL, Wei JY (1997). Increased gait unsteadiness in community-dwelling elderly fallers. Arch Phys Med Rehabil.

[B20] Shumway-Cook A, Baldwin M, Polissar NL, Gruber W (1997). Predicting the probability for falls in community-dwelling older adults. Phys Ther.

[B21] Spirduso WW, Spirduso WW (1995). Balance, posture and locomotion: Physical Dimensions of Aging.

[B22] Stelmach GE, Worringham CJ (1985). Sensorimotor deficits related to postural stability. Implications for falling in the elderly. Clin Geriatr Med.

[B23] Tinetti ME, Speechley M, Ginter SF (1988). Risk factors for falls among elderly persons living in the community. N Engl J Med.

[B24] Mulder T, Zijlstra W, Geurts A (2002). Assessment of motor recovery and decline. Gait Posture.

[B25] Wickens CD, Parasuraman R, Davies DR (1984). Processing resources in attention: Varieties of Attention.

[B26] de Visser E, Veth RP, Schreuder HW, Duysens J, Mulder T (2003). Reorganization of gait after limb-saving surgery of the lower limb. Am J Phys Med Rehabil.

[B27] Huitema RB, Hof AL, Mulder T, Brouwer WH, Dekker R, Postema K (2004). Functional recovery of gait and joint kinematics after right hemispheric stroke. Arch Phys Med Rehabil.

[B28] Bergland A, Pettersen AM, Laake K (1998). Falls reported among elderly Norwegians living at home. Physiother Res Int.

[B29] Moe-Nilssen R, Nordin E, Lundin-Olsson L (2008). Work Package 3 of European Community Research Network Prevention of Falls Network Europe. Criteria for evaluation of measurement properties of clinical balance measures for use in fall prevention studies. J Eval Clin Pract.

[B30] Lamb SE, Jørstad-Stein EC, Hauer K, Becker C (2006). Prevention of Falls Network Europe and Outcomes Consensus Group. Development of a common outcome data set for fall injury prevention trials: the Prevention of Falls Network Europe consensus. J Am Geriatr Soc.

[B31] Gibson MJ, Andres RO, Isaacs B, Radebaugh T, Worm-Petersen J (1987). The prevention of falls in later life. A report of the Kelloggs International Work Group. Dan Med Bull.

[B32] AMTI Balance Clinic (2001). Users Manual Version 11.

[B33] Le Clair K, Riach C (1996). Postural stability measures: what to measure and for how long. Clin Biomech.

[B34] Jamet M, Deviterne D, Gauchard GC, Vancon G, Perrin PP (2007). Age-related part taken by attentional cognitive processes in standing postural control in a dual-task context. Gait Posture.

[B35] Pajala S, Era P, Koskenvuo M, Kaprio J, Tolvanen A, Rantanen T (2007). Genetic and environmental contribution to postural balance of older women in single and dual task situations. Neurobiol Aging.

[B36] Pellecchia GL (2005). Dual-task training reduces impact of cognitive task on postural sway. J Mot Behav.

[B37] Andersson G, Hagman J, Talianzadeh R, Svedberg A, Larsen HC (2002). Effect of cognitive load on postural control. Brain Res Bull.

[B38] Lezak MD (2004). Neuropsychological assessment.

[B39] de Vet HCW, Terwee CB, Knol DL, Bouter LM (2006). When to use agreement versus reliability measures. J Clin Epidemiol.

[B40] Smidt N, Windt DA van der, Assendelft WJ, Mourits AJ, Deville WL, de Winter AF, Bouter LM (2002). Interobserver reproducibility of the assessment of severity of complaints, grip strength, and pressure pain threshold in patients with lateral epicondylitis. Arch Phys Med Rehabil.

[B41] Streiner DL, Norman GR (2003). Health measurement scales: A practical guide to their development and use.

[B42] Portney LG, Watkins MP (1993). Foundations of Clinical Research Applications to Practice.

[B43] Shrout PE, Fleiss JL (1979). Intraclass correlation: uses in assessing rater reliability. Psychological Bulletin.

[B44] Roebroeck ME, Harlaar J, Lankhorst GJ (1993). The application of generalizability theory to reliability assessment: an illustration using isometric force measurements. Phys Ther.

[B45] Stratford P (1989). Reliability: consistency of differentiating among subjects (letter). Phys Ther.

[B46] Bland JM, Altman DG (1986). Statistical methods for assessing agreement between two methods of clinical measurement. Lancet.

[B47] Day BL, Steiger MJ, Thompson PD, Marsden CD (1993). Effect of vision and stance width on human body motion when standing: implications for afferent control of lateral sway. J Physiol.

[B48] Sims KJ, Brauer SG (2000). A rapid upward step challenges medio-lateral postural stability. Gait Posture.

[B49] Kirby RL, Price NA, MacLeod DA (1987). The influence of foot position on standing balance. J Biomech.

[B50] Chiari L, Rocchi L, Cappello A (2002). Stabilometric parameters are affected by anthropometry and foot placement. Clin Biomech.

