# Supplementary material for: The reliability of postural balance measures in single and dual tasking in elderly fallers and non-fallers
Source: BMC Musculoskelet Disord. 2008 Dec 9;9:162. doi: 10.1186/1471-2474-9-162 (PMC2614424; doi:10.1186/1471-2474-9-162)
Supplement: Additional file 1 — Bland and Altman plots of the measured balance variables. The data provided represent the analysis with Bland-Altman plots of the measured balance variables. [file 1471-2474-9-162-S1.doc]

Appendix I

Figure 1. Bland and Altman plots of the maximal displacement in medial-lateral direction (Max-ML) in single and dual-task conditions, with and without vision.

| a) Single task with vision | b) Dual task with vision |
| --- | --- |
| 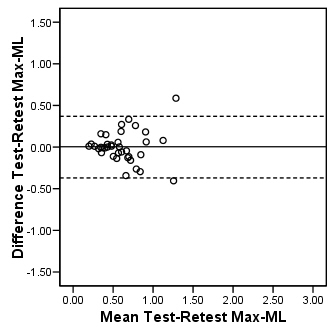 | 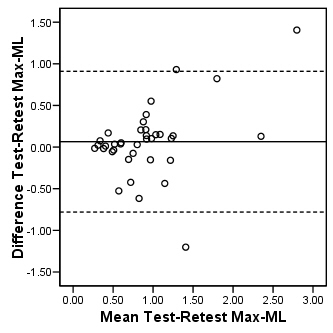 |
| c) Single task no vision | d) Dual task no vision |
| 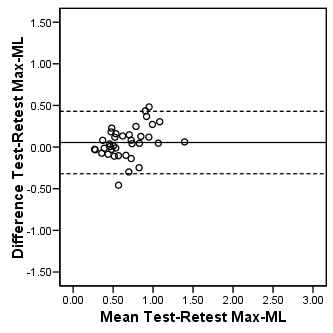 | 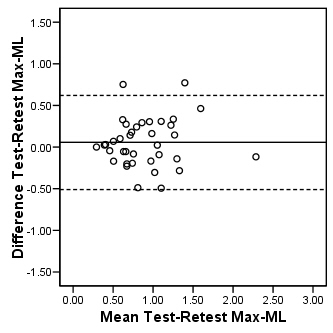 |

Figure 2. Bland and Altman plots of the root mean square of the maximal displacement in medial-lateral direction (RMS-ML) in single and dual-task conditions, with and without vision.

| a) Single task with vision | b) Dual task with vision |
| --- | --- |
| 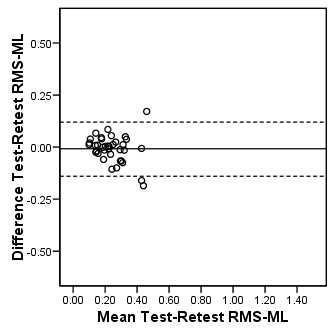 | 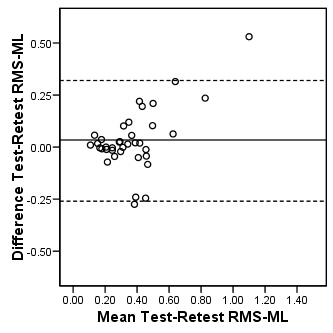 |
| c) Single task no vision | d) Dual task no vision |
| 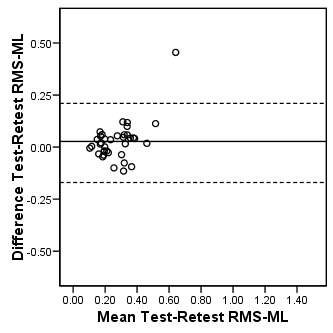 | 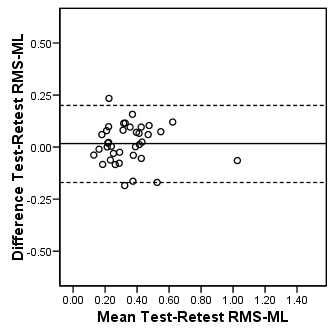 |

Figure 3. Bland and Altman plots of the maximal displacement in anterior-posterior direction (Max-AP) in single and dual-task conditions, with and without vision.

| a) Single task with vision | b) Dual task with vision |
| --- | --- |
| 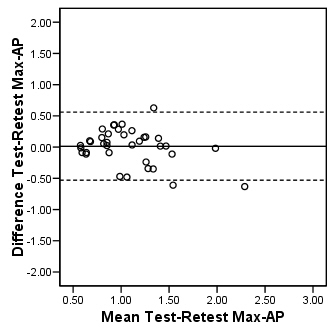 | 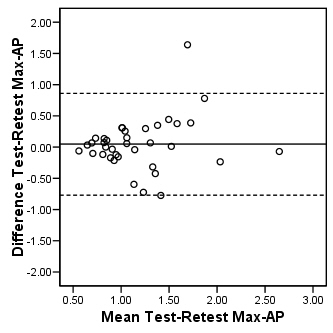 |
| c) Single task no vision | d) Dual task no vision |
| 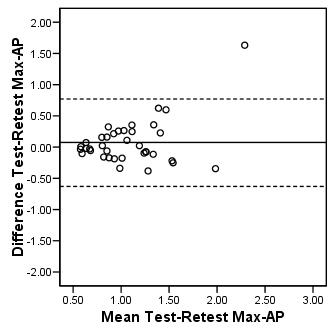 | 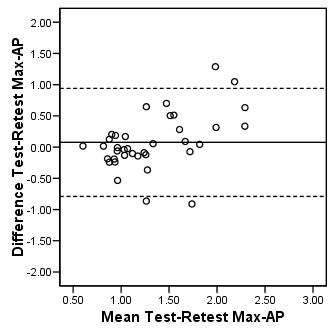 |

Figure 4. Bland and Altman plots of the root mean square of the maximal displacement in anterior-posterior direction (RMS-AP) in single and dual-task conditions, with and without vision.

| a) Single task with vision | b) Dual task with vision |
| --- | --- |
| 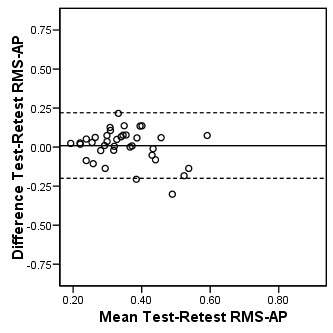 | 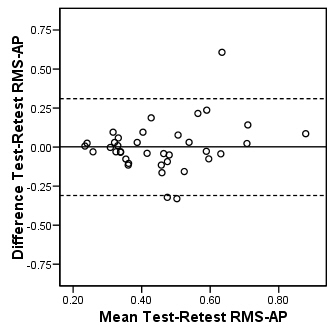 |
| c) Single task no vision | d) Dual task no vision |
| 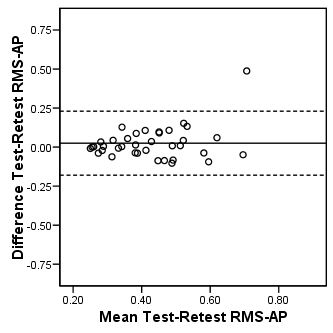 | 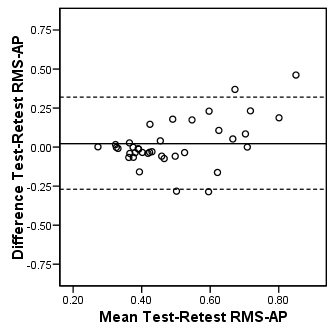 |

Figure 5. Bland and Altman plots of the average speed of displacement (MV) in single and dual-task conditions, with and without vision.

| a) Single task with vision | b) Dual task with vision |
| --- | --- |
| 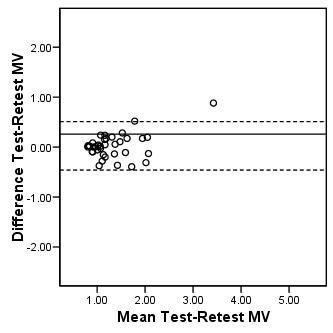 | 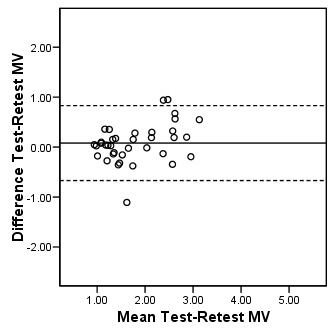 |
| c) Single task no vision | d) Dual task no vision |
| 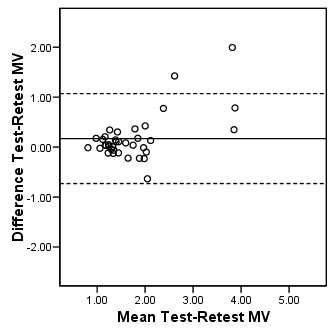 | 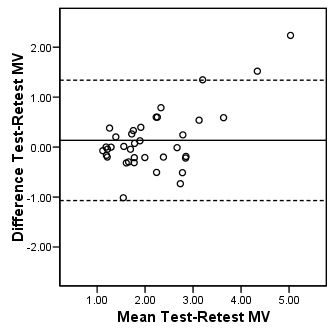 |

Figure 6. Bland and Altman plots of the area of the 95^th^ percentile ellipse (AoE) in single and dual-task conditions, with and without vision.

| a) Single task with vision | b) Dual task with vision |
| --- | --- |
| 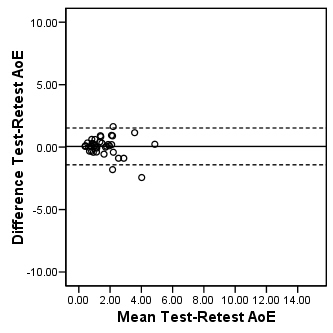 | 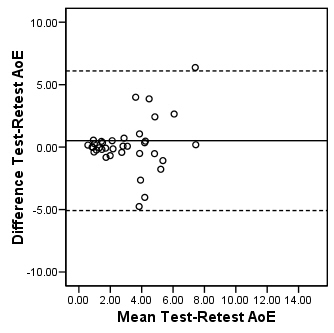 |
| c) Single task no vision | d) Dual task no vision |
| 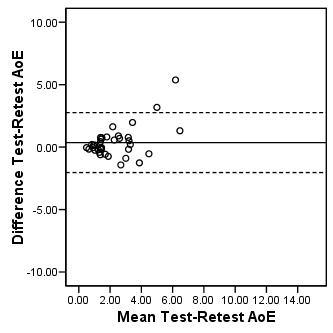 | 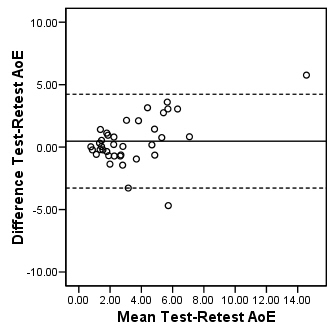 |
